# A Multi-Enzyme Cascade Reaction for the Production of 2′3′-cGAMP

**DOI:** 10.3390/biom11040590

**Published:** 2021-04-16

**Authors:** Martin Becker, Patrick Nikel, Jennifer N. Andexer, Stephan Lütz, Katrin Rosenthal

**Affiliations:** 1 Chair for Bioprocess Engineering, Department of Biochemical and Chemical Engineering, TU Dortmund University, D-44227 Dortmund, Germany; martin4.becker@tu-dortmund.de (M.B.); patrick.nikel@tu-dortmund.de (P.N.); stephan.luetz@tu-dortmund.de (S.L.); 2 Institute of Pharmaceutical Sciences, University of Freiburg, D-79104 Freiburg, Germany; jennifer.andexer@pharmazie.uni-freiburg.de

**Keywords:** enzyme cascade, polyphosphate kinase, multi-step reaction, ATP, 2′3′-cGAMP, cGAS, cyclic dinucleotide (CDN), in vitro biotransformation, biocatalysis

## Abstract

Multi-enzyme cascade reactions for the synthesis of complex products have gained importance in recent decades. Their advantages compared to single biotransformations include the possibility to synthesize complex molecules without purification of reaction intermediates, easier handling of unstable intermediates, and dealing with unfavorable thermodynamics by coupled equilibria. In this study, a four-enzyme cascade consisting of *Sc*ADK, *Aj*PPK2, and *Sm*PPK2 for ATP synthesis from adenosine coupled to the cyclic GMP-AMP synthase (cGAS) catalyzing cyclic GMP-AMP (2′3′-cGAMP) formation was successfully developed. The 2′3′-cGAMP synthesis rates were comparable to the maximal reaction rate achieved in single-step reactions. An iterative optimization of substrate, cofactor, and enzyme concentrations led to an overall yield of 0.08 mole 2′3′-cGAMP per mole adenosine, which is comparable to chemical synthesis. The established enzyme cascade enabled the synthesis of 2′3′-cGAMP from GTP and inexpensive adenosine as well as polyphosphate in a biocatalytic one-pot reaction, demonstrating the performance capabilities of multi-enzyme cascades for the synthesis of pharmaceutically relevant products.

## 1. Introduction

Nature efficiently synthesizes metabolites with sequential multi-step enzyme systems, which are part of the complex metabolic network. In whole-cell biocatalysis, the natural metabolic network of microorganisms is exploited to produce valuable products from inexpensive substrates. In in vitro enzyme cascades, the cell’s natural metabolism is dispensed with, so that consecutive reactions can be adapted to produce relevant chemicals by smart selection of the biocatalysts. As these enzyme cascade reactions offer many advantages, they have gained importance in recent years [1]. Higher yields can be achieved and the isolation and purification of intermediates is not necessary, which results in the minimization of waste and costs [2]. Furthermore, enzyme cascades offer the opportunity to deal with unstable or toxic intermediates, as they do not accumulate and are further converted into the final product [3]. They can also be used to shift the equilibrium of thermodynamically unfavorable reactions to increase the reaction yield [4].

However, exploiting these advantages of enzyme cascades requires extensive adaptation and optimization to circumvent incompatibility issues between the individual reactions. A suitable pH, reaction temperature and buffer must be found at which all enzymes are active [5]. The concentrations of the enzymes and substrates must be adjusted to each other in a suitable manner in order to avoid side reactions and inhibitory effects and to achieve a balanced flux through the reaction cascade without accumulation of intermediates [6,7]. Outstanding examples for successfully applied enzyme cascades are the synthesis of norpseudoephedrine and norephedrine from the cheap substrates benzaldehyde and pyruvate by only two enzymes [8]. Moreover, the synthesis of ε-caprolactone from cyclohexanone and 1,6-hexanediol with an enzyme cascade consisting of a Baeyer-Villiger monooxygenase and an alcohol dehydrogenase shows successful optimization resulting in a conversion of 99% [9]. Conversions of up to 96% were achieved for the synthesis of fatty amines from fatty acids using a carboxylic acid reductase and ω-transaminase [10].

**Figure 1:**
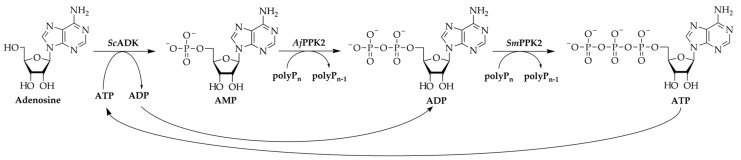
Enzyme cascade for the regeneration of ATP from adenosine using polyphosphate (polyP) as a phosphate donor. The cascade consists of the three enzymes adenosine kinase from *Saccharomyces cerevisiae* (*Sc*ADK), PPK2 from *Acinetobacter johnsonii* (*Aj*PPK2), and PPK2 from *Sinorhizobium meliloti* (*Sm*PPK2). Adenosine 5′-monophosphate (AMP), adenosine 5′-diphosphate (ADP), adenosine 5′-triphosphate (ATP).

A limitation of enzyme cascades is in the supply of group-transferring cofactors such as nicotinamides or nucleoside triphosphates, which are consumed in stoichiometric quantities [11]. A variety of regeneration systems has been developed to enable an efficient regeneration of these expensive compounds. In the case of adenosine 5′-triphosphate (ATP), acetate kinases in combination with acetyl phosphate, or pyruvate kinase with phosphoenolpyruvate as substrate are commonly used [12,13]. Additionally, polyphosphate kinases 2 (PPK2) have gained increased interest, which require the cheap phosphate donor polyphosphate (polyP) for phosphorylation of nucleotides [14]. In combination with an adenosine kinase, PPK2s were used to regenerate ATP for an *S*-adenosylmethionine regeneration cascade [15,16]. This ATP regeneration cascade consists of the adenosine kinase from *Saccharomyces cerevisiae* (*Sc*ADK), a polyphosphate kinase from *Acinetobacter johnsonii* (*Aj*PPK2), and a polyphosphate kinase from *Sinorhizobium meliloti* (*Sm*PPK2) (Figure 1).

The *Sc*ADK catalyzes the transfer of γ-phosphate of ATP on adenosine synthesizing adenosine 5′-monophosphate (AMP) with stoichiometric ATP dephosphorylation. The *Aj*PPK2 subsequently catalyzes the phosphorylation of AMP into adenosine 5′-diphosphate (ADP) with polyP as phosphate donor. In the last step, *Sm*PPK2 phosphorylates ADP into ATP. So far, the cascade has mainly been used for the regeneration of ATP from adenosine or in a shortened form from AMP [15,17]. However, the enzyme cascade is not only relevant for regeneration but might also be applicable for the synthesis of ATP for ATP-consuming reactions, since nucleosides are generally cheaper and more stable than nucleotides [18].

A highly relevant biocatalyst, which has been investigated recently, is the cyclic GMP-AMP synthase (cGAS) requiring ATP and guanosine 5′-triphosphate (GTP) as substrates for the synthesis of 2′3′-cyclic GMP-AMP (2′3′-cGAMP) [19]. The catalytic activity of cGAS for 2′3′-cGAMP synthesis is induced in the presence of cytosolic DNA [20]. The second messenger 2′3′-cGAMP triggers a signaling cascade upon binding to the stimulator of interferon gene receptors (STING) at the endoplasmic reticulum, which activates the innate immune system through the release of interferons. These properties make 2′3′-cGAMP an interesting candidate in drug discovery, e.g., for cancer immunotherapy or as an adjuvant in vaccines [21,22]. The biocatalytic synthesis of 2′3′-cGAMP using cGAS and nucleoside 5′-triphosphates as starting material has already been extensively investigated [23,24].

In this study, we developed a four-enzyme cascade consisting of three enzymes for ATP synthesis coupled with truncated human cGAS (thscGAS) for 2′3′-cGAMP production. All required enzymes were characterized with regard to their specific activities in order to balance their concentrations for subsequent cascade development. Additionally, enzyme and substrate concentrations were optimized to ensure sufficient ATP supply. Finally, the enzyme cascade consisting of *Sc*ADK, *Aj*PPK2, *Sm*PPK2, and thscGAS was successfully established for the synthesis of 2′3′-cGAMP from adenosine and GTP. The achieved synthesis rate and final concentration of 2′3′-cGAMP were comparable to experiments with ATP as substrate. This demonstrated the first approach of an enzyme cascade for 2′3′-cGAMP synthesis from adenosine.

## 2. Materials and Methods

### 2.1. Plasmids and Strains

The kinases were produced with *E. coli* BL21 (DE3) pLysS pET28a-*ScADK*, *E. coli* BL21 (DE3) pET28a-*AjPPK2* and *E. coli* BL21 (DE3) pET28a-*SmPPK2*. Descriptions of the plasmids for the expression of *Sc*ADK, *Aj*PPK2 and *Sm*PPK2 can be found in [15]. The thscGAS expression strain *E. coli* BL21 (DE3) pLysS pET28a*-SUMOthscGAS* is described in [24].

### 2.2. Recombinant Enzyme Expression

The chemicals used for enzyme expression were purchased from VWR (VWR International GmbH, Darmstadt, Germany), ThermoFisher (ThermoFisher Scientific, Waltham, MA, USA) and Roth (Carl Roth, Karlsruhe, Germany). The strains were spread on LB (10 g L^−1^ tryptone, 5 g L^−1^ yeast extract, 5 g L^−1^ NaCl) agar plates containing 50 mg L^−1^ kanamycin. For strains containing the pLysS plasmid, 25 mg L^−1^ chloramphenicol were added. Agar plates were incubated over night at 37 °C. The next day, a pre-culture was inoculated from one colony in 10 mL LB medium in case of the expression of the kinases and 10 mL 2xYT (16 g L^−1^ tryptone, 10 g L^−1^ yeast extract, 5 g L^−1^ NaCl) medium in case of thscGAS, using the same antibiotic concentrations as used for agar plates. The pre-cultures were incubated for 8 h at 37 °C and 200 rpm. For the main cultures, the same media compositions were used as in the pre-cultures and were inoculated to an OD_600_ of 0.05 in case of the expression of the kinases and OD_600_ of 0.1 for thscGAS. Here, 200 mL of culture were incubated in a 2-L baffled shaking flask in the case of thscGAS and 400 mL in the case of the expression of the kinases. The main cultures were incubated at 37 °C and 200 rpm. After an OD_600_ of 1 was reached, enzyme expression was induced with 0.5 mM isopropyl-β-D-thiogalactopyranosid (IPTG). Subsequently, the main cultures were incubated at 20 °C for 11 h in case of thscGAS and overnight in case of kinases. The cells were harvested by centrifugation (25 min, 4 °C, 4700× *g*) in 50 mL aliquots and cell pellets were stored at −20 °C.

### 2.3. Enzyme Purification

The chemicals used for enzyme purification were purchased from Roth (Carl Roth, Karlsruhe, Germany), Merck (Merck KGaA, Darmstadt, Germany), VWR (VWR International GmbH, Darmstadt, Germany) and AppliChem (AppliChem GmbH, Darmstadt, Germany) in the highest available purity. Two cell pellets were resuspended in 20 mL lysis buffer (kinases: 40 mM TRIS-HCl, 100 mM NaCl, 10% (*v*/*v*) glycerol, pH 8; thscGAS: 50 mM TRIS-HCl, 300 mM NaCl, 40 mM imidazole, 1 mM tris(2-carboxyethyl)phosphine (TCEP), pH 8.0). Cells were disrupted by sonication in five cycles on ice using a Branson Digital Sonifier (BRANSON Ultrasonics Corporation, Danbury, CT, USA). The cycle time was 30 s with a pulse on time of 0.5 s and a pulse off time of 1 s. The sonication amplitude was 10%. By centrifugation (20 min, 4 °C, 43,000× *g*), insoluble cellular components were removed, and the supernatant was sterile filtered (0.2 µm). The filtrate was loaded onto a 1 mL HisTrap™ FF crude column (GE Healthcare, Solingen, Germany), whereby the column was previously equilibrated with 5 column volumes (CV) of ultrapure water and 10 CV of lysis buffer. After washing the column with 10 CV lysis buffer, the enzyme was eluted in six fractions of 1 mL with elution buffer (20 mM TRIS-HCl, 150 mM NaCl, 300 mM imidazole, pH 7.4). The fractions with the highest protein concentrations were identified by Bradford assay and combined to a total volume of 2.5 mL. Interfering imidazole was removed by a PD-10 column (GE Healthcare, Solingen, Germany), which was previously equilibrated with 25 mL ultrapure water and 25 mL buffer (50 mM TRIS-HCl, 40 mM MgCl_2_ · 6H_2_O, pH 8.0). The enzyme was eluted in 3.5 mL buffer. The final enzyme concentration was determined by a Bradford assay. SDS gels of all enzyme purifications can be found in the Supplementary Materials (Figures S1–S5).

### 2.4. Enzyme Cascade Reactions

The chemicals used for the enzyme cascade reactions were purchased from Roth (Carl Roth, Karlsruhe, Germany), AppliChem (AppliChem GmbH, Darmstadt, Germany), Acros Organics (ThermoFisher Scientific, Waltham, MA, USA) and Merck (Merck KGaA, Darmstadt, Germany) in the highest available purity. The enzyme cascade reactions were performed in 50 mM TRIS-HCl buffer in a 2 mL microtube, with a reaction volume of 1 mL. The concentrations of the substrates adenosine, AMP, ADP, ATP, GTP were varied between 0.5 and 10 mM as indicated. The polyP concentration was 30 mM, whereby the concentration was calculated based on the molecular weight of 101.96 g mol^−1^ for a single phosphate monomer. The enzyme concentrations of the kinases were varied as indicated between 0.5 and 50 mg L^−1^ and the concentration of thscGAS between 40 and 120 mg L^−1^. Additionally, 0.1 mg mL^−1^ herring testis DNA (HT DNA) was added for thscGAS activation. The reaction was started by adding the substrate and samples were incubated at 37 °C and 300 rpm in an orbital shaker for 24 h. Sample times: 0 min, 35 min, 45 min, 1 h, 2 h, 3 h, 4 h, 5 h, 18 h, 21 h, and 24 h. The reaction was stopped by heating the samples to 95 °C for 5 min. Reaction rates and specific activities for 2′3′-cGAMP synthesis were determined from 2′3′-cGAMP concentrations in the first 5 h of reaction.

### 2.5. In Vitro Enzyme Assays

The chemicals used for in vitro enzyme assays were purchased from Roth (Carl Roth, Karlsruhe, Germany), AppliChem (AppliChem GmbH, Darmstadt, Germany), Acros Organics (ThermoFisher Scientific, Waltham, MA, USA) and Merck (Merck KGaA, Darmstadt, Germany) in the highest available purity. The enzyme assays were performed in the 50 mM TRIS-HCl buffer in 2 mL microtubes. The concentrations of the substrates adenosine, AMP, ADP, and ATP were 10 mM. The polyP concentration was 15 mM, whereby the concentration was calculated as described above. The enzyme concentrations of the kinases were 5 mg L^−1^ in the case of *Sc*ADK and *Sm*PPK2 and 0.5 mg L^−1^ in the case of the *Aj*PPK2. The reaction was started by adding the substrates and incubated at 37 °C and 300 rpm in an orbital shaker for 2.5 h. Samples were taken after 0, 15, 30, 45, 60, 75, 90, 120, and 150 min. The reaction was stopped by heating the samples to 95 °C for 5 min. Specific activities were determined from product concentrations in the first 2 to 2.5 h.

### 2.6. Quantification of 2′3′-cGAMP, Adenosine, AMP, ADP, ATP, and GTP

The chemicals used for the analysis were purchased from VWR (VWR International GmbH, Darmstadt, Germany) and Roth (Carl Roth, Karlsruhe, Germany) in the highest available purity. The biotransformation samples were analyzed by reversed-phase high performance liquid chromatography (RP-HPLC). A Knauer Azura HPLC system (KNAUER GmbH, Berlin, Germany) consisting of a pump (P 6.1L), autosampler (AS 6.1L), column oven (CT 2.1), and a wavelength detector (MWD 2.1L). The system was equipped with an ISAspher 100-5 C18 AQ column (250 × 4 mm, Isera, Düren, Germany) heated to 30 °C. The gradient used for the analysis consisted of mobile phase A (0.06 M K_2_HPO_4_ and 0.04 M KH_2_PO_4_) and mobile phase B (95% acetonitrile, 5% water) at a flow rate of 1 mL min^−1^ (0 min: 100% A; 3 min: 100% A; 10.5 min: 82% A, 18% B; 16 min: 50% A, 50% B; 18 min: 100% A; 23 min: 100% A). The analytes were detected at a wavelength of 254 nm. The chromatograms were analyzed using ClarityChrom (KNAUER GmbH, Berlin, Germany).

## 3. Results and Discussion

### 3.1. 2′3′-cGAMP Synthesis by a Multi-Enzyme Cascade Reaction

Enzyme cascades have been widely used for in vitro cofactor regeneration. Polyphosphate kinases were found to be suitable for ATP regeneration, as they require inexpensive polyP for the phosphorylation of AMP to ADP and ADP to ATP, respectively. The chosen kinases *Sc*ADK, *Aj*PPK2, and *Sm*PPK2 are a well-established regeneration system of ATP [11] that was now extended with the enzyme thscGAS, which catalyzes the cyclization of ATP and GTP for 2′3′-cGAMP synthesis [24].

**Figure 2:**
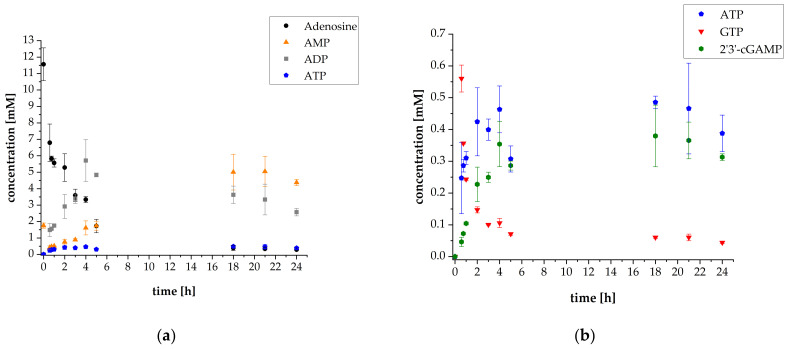
Reaction progress of enzyme reaction cascade for 2′3′-cGAMP synthesis (used enzyme concentrations: 50 mg L^−1^ *Sc*ADK, 50 mg L^−1^ *Aj*PPK2, 50 mg L^−1^ *Sm*PPK2 and 40 mg L^−1^ thscGAS): (**a**) Mean adenosine, AMP, ADP, and ATP concentrations; (**b**) Mean ATP, GTP, and 2′3′-cGAMP concentrations. Enzyme assays were performed in 50 mM TRIS-HCl buffer containing 40 mM MgCl_2_ · 6H_2_O, pH 8.0. The reaction volume was 1 mL, which was incubated at 37 °C and 300 rpm in an orbital shaker. Substrate concentrations: 10 mM adenosine, 1 mM AMP, and 30 mM polyP calculated as single phosphate unit. Addition after 35 min: 0.5 mM GTP, 0.1 mg mL^−1^ HT DNA, and 40 mg L^−1^ thscGAS. Sample times: 0 min, 35 min, 45 min, 1 h, 2 h, 3 h, 4 h, 5 h, 18 h, 21 h, and 24 h. Error bars relate to biological triplicates.

The initial cascade reaction conditions were set according to Mordhorst et al. [15]. The concentrations of *Sc*ADK, *Aj*PPK2, and *Sm*PPK2 were 50 mg L^−1^. The initial substrate concentrations were 10 mM adenosine and 30 mM polyP calculated as single phosphate units. Additionally, 1 mM AMP was added to start the cascade reaction. In order to provide sufficient amounts of intermediates for the subsequent reaction step, the synthesis of 2′3′-cGAMP was started after 35 min by adding thscGAS, 0.5 mM GTP as the second substrate and 0.1 mg mL^−1^ HT DNA, required for thscGAS activation. A thscGAS concentration of 40 mg L^−1^ was chosen, as this concentration was commonly used in previously established assays [24,25]. The results of the reaction progress of the first thscGAS coupled cascade experiment are shown in Figure 2.

The ATP synthesis cascade was successfully coupled to the 2′3′-cGAMP-producing enzyme thscGAS. The final 2′3′-cGAMP concentration was 0.31 ± 0.01 mM, which is in the concentration range usually obtained with this enzyme. The specific activity of thscGAS was 0.052 ± 0.014 U mg^−1^ (Figure S6), which is in the same order of magnitude compared to a specific activity of 0.073 ± 0.008 U mg^−1^ that was obtained in assays with isolated thscGAS with ATP and GTP as substrates [25]. Adenosine was almost consumed with a final concentration of 0.29 mM. The GTP concentration decreased from 0.56 ± 0.04 mM to 0.04 ± 0.01 mM after 24 h. Interestingly, more GTP was consumed than 2′3′-cGAMP was synthesized. This suggests that GTP is also involved in other reactions in the cascade and might serve as a phosphate donor for AMP synthesis by the *Sc*ADK. Up to now, the GTP acceptance as phosphate donor of *Sc*ADK-catalyzed reactions has not been investigated. However, it is generally known that adenosine kinases accept GTP as phosphate donor as shown for an adenosine kinase from *Trypanosoma brucei* with K_M_ values of 26 ± 2.4 μmol L^−1^ for GTP and 82 ± 14 μmol L^−1^ for ATP, respectively [26].

Nevertheless, even though GTP was not fully converted into 2′3′-cGAMP, the obtained results are very valuable because thscGAS has never been investigated in such a complex reaction system, opening the door to further studies. In particular, the presence of a high concentration of phosphate-group containing substrates was expected to be critical, as it is known that polyP complexes cations [27]. Metal ions, in this case Mg^2+^, are essential for the catalytic activity of thscGAS, as well as for the kinases. Limitations caused by Mg^2+^ complexation by polyP have to be prevented by balancing the polyP level to an optimal concentration. A concentration of up to 30 mM has been described as non-inhibitory in previous studies for the kinases and also seems to be tolerated by thscGAS [27].

### 3.2. Specific Activities of the Kinases

The specific activities of the kinases *Sc*ADK, *Aj*PPK2, and *Sm*PPK2 were determined in order to adjust the amounts of enzymes in the cascade with the goal to increase the 2′3′-cGAMP synthesis rate, yield and enzyme usage (amount of product per amount of enzyme). Purified *Sc*ADK was tested with 10 mM adenosine and 10 mM ATP; purified *Aj*PPK2 and *Sm*PPK2 were tested with 10 mM AMP and ADP, respectively, as well as 15 mM polyP calculated as single phosphate units. All assays were performed in a 50 mM TRIS-HCl buffer containing 40 mM MgCl_2_ · 6 H_2_O at pH 8. The enzyme concentrations were adjusted to obtain a linear correlation of the product concentration increase as a function of time for the calculation of reaction rates. The reaction progress was monitored for 2.5 h. The determined reaction rates and specific activities are summarized in Table 1 (Figures S9–S11).

The *Sc*ADK had a specific activity of 4.3 ± 0.9 U mg^−1^, which is substantially lower compared to the published values of about 100 U mg^−1^ [28]. The decreased specific activity might be caused by the His-tag, which was omitted in the published study. Although it is generally assumed that the His-tag has minimal or no effect on the functionality or structure of most of proteins, a negative impact cannot be excluded, as a few studies revealed the influence of the His-tag on the properties such as a decrease in the enzyme’s activity [29,30]. The highest specific activity of the three kinases was determined for the *Aj*PPK2 with 69.2 U mg^−1^. Interestingly, the specific activity was substantially higher than the already published values of about 0.076 U mg^−1^ [31], which might be caused by differences in assay conditions, such as higher polyP and substrate concentrations in this study. The enzyme *Sm*PPK2 had a specific activity of 2.8 ± 0.6 U mg^−1^, which is the first published reaction rate of this enzyme. The obtained specific activity is in the range of *Ft*PPK2 class 1 [32], but is substantially lower than the recently reported specific activities of *Hb*PPK2 (189 ± 13 U mg^−1^) and *Nd*PPK2 (124 ± 20 U mg^−1^) [33]. In further optimization steps, it could be investigated whether *Sm*PPK2 can be replaced by one of these enzymes in order to achieve higher reaction rates and yields with lower enzyme usage.

### 3.3. Adaptation of the Assay Composition in the Multi-Enzyme Cascade Reaction

**Figure 3:**
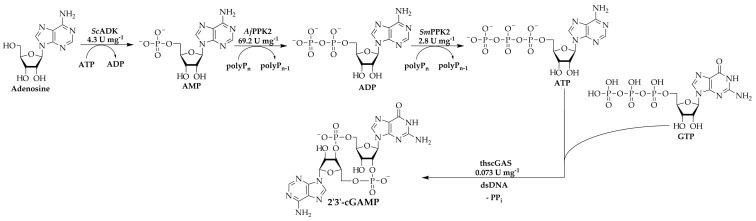
2′3′-cGAMP synthesis using a multi-enzyme cascade reaction. The cascade consists of *Sc*ADK, *Aj*PPK2, *Sm*PPK2, and thscGAS with indicated specific activities quantified in individual single-step reactions with isolated enzyme. The specific activity of thscGAS was taken from Rolf et al. [25].

With the determined specific enzyme activities, the ratio of enzyme concentrations was adjusted to increase the 2′3′-cGAMP synthesis rate, yield and enzyme usage. Figure 3 gives an overview of the multi-enzyme cascade reaction and specific activities of all enzymes involved.

**Figure 4:**
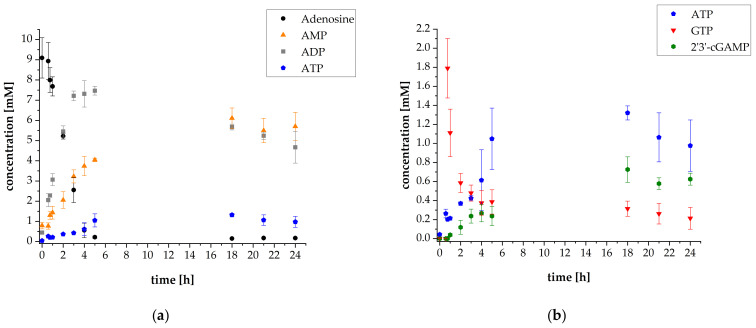
Reaction progress of enzyme reaction cascade for 2′3′-cGAMP synthesis (used enzyme concentrations: 50 mg L^−1^ *Sc*ADK, 5 mg L^−1^ *Aj*PPK2, 50 mg L^−1^ *Sm*PPK2 and 120 mg L^−1^ thscGAS): (**a**) Mean adenosine, AMP, ADP, and ATP concentrations; (**b**) mean ATP, GTP, and 2′3′-cGAMP concentrations. Enzyme assays were performed in 50 mM TRIS-HCl buffer containing 40 mM MgCl_2_ · 6H_2_O, pH 8.0. The reaction volume was 1 mL, which was incubated at 37 °C and 300 rpm in an orbital shaker. Substrate concentrations: 10 mM adenosine, 1 mM AMP, and 30 mM polyP calculated as single phosphate units. Addition after 35 min: 2.4 mM GTP, 0.1 mg mL^−1^ HT DNA, and 120 mg L^−1^ thscGAS. Sample times: 0 min, 35 min, 45 min, 1 h, 2 h, 3 h, 4 h, 5 h, 18 h, 21 h, and 24 h. Error bars relate to biological triplicates.

Since the specific activity of thscGAS is only 0.073 U mg^−1^ [25], the concentration was increased from 40 mg L^−1^ to 120 mg L^−1^. In turn, the concentration of *Aj*PPK2 with a specific activity of 69.2 ± 10.8 U mg^−1^, was decreased to 5 mg L^−1^. Furthermore, the concentration of GTP was increased from 0.5 mM to 2.4 mM to decrease limitations of 2′3′-cGAMP synthesis by enhanced GTP availability. Higher GTP concentrations were not tested because the thscGAS-catalyzed synthesis of 2′3′-cGAMP is inhibited by greater substrate concentrations [34]. Figure 4 shows the reaction progress.

The variations in enzyme and substrate concentrations resulted in a substantially higher final 2′3′-cGAMP concentration of 0.63 ± 0.06 mM corresponding to a twofold increase. In contrast, the specific thscGAS activity decreased to 0.012 ± 0.004 U mg^−1^ (Figure S7). The GTP concentration decreased from 1.79 ± 0.31 mM to 0.21 ± 0.11 mM within 24 h, which implies that almost twice as much GTP was consumed as 2′3′-cGAMP was synthesized. GTP dephosphorylation as cofactor for the *Sc*ADK-catalyzed AMP synthesis is also assumed here. Lowering the *Aj*PPK2 concentration did not negatively affect the ATP supply for 2′3′-cGAMP synthesis. In contrast, the accumulated ATP concentration of 0.98 ± 0.27 mM after 24 h was 1.5-fold higher compared to the previous reaction cascade. Adenosine was almost depleted with a final concentration of 0.18 ± 0.02 mM after 24 h. The reason for the reaction progress stagnation remains to be investigated, as the substrates ATP and GTP were not completely consumed and could be converted into 2′3′-cGAMP. Stability issues may be a possible reason for this. An enzyme usage of 1.87 ± 0.19 mg_2′3′-cGAMP_ mg_enzyme_^−1^ was calculated for this experiment, which was substantially higher compared to the previous experiment with 1.12 ± 0.04 mg_2′3′-cGAMP_ mg_enzyme_^−1^.

**Figure 5:**
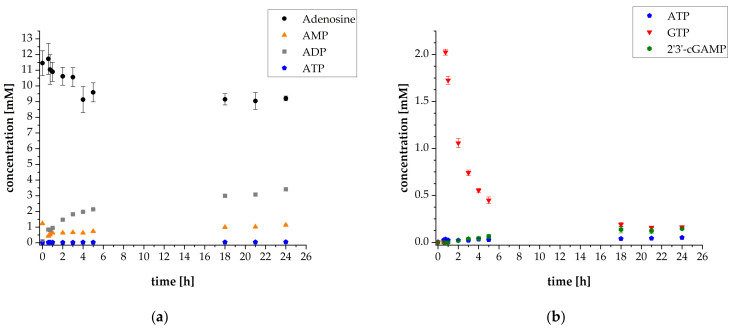
Reaction progress of enzyme reaction cascade for 2′3′-cGAMP synthesis (used enzyme concentrations: 5 mg L^−1^ *Sc*ADK, 0.5 mg L^−1^ *Aj*PPK2, 5 mg L^−1^ *Sm*PPK2 and 120 mg L^−1^ thscGAS): (**a**) Mean adenosine, AMP, ADP and ATP concentrations; (**b**) Mean ATP, GTP and 2′3′-cGAMP concentrations. Enzyme assays were performed in 50 mM TRIS-HCl buffer containing 40 mM MgCl_2_ · 6H_2_O, pH 8.0. The reaction volume was 1 mL, which was incubated at 37 °C and 300 rpm in an orbital shaker. Substrate concentrations: 10 mM adenosine, 1 mM AMP, and 30 mM polyP. Addition after 35 min: 2.4 mM GTP, 0.1 mg mL^−1^ HT DNA, and 120 mg L^−1^ thscGAS. Sample times: 0 min, 35 min, 45 min, 1 h, 2 h, 3 h, 4 h, 5 h, 18 h, 21 h, and 24 h. Error bars relate to biological duplicates.

To further increase enzyme utilization, the concentrations of the kinases were decreased. As the enzyme thscGAS has a comparatively low specific activity, no limitation of ATP availability was expected. The concentrations of the kinases were reduced by one tenth compared to the previous experiment. The enzyme cascade thus consisted of 5 mg L^−1^ *Sc*ADK as well as 5 mg L^−1^ *Sm*PPK2 and 0.5 mg L^−1^ *Aj*PPK2. The concentration of thscGAS was maintained at 120 mg L^−1^. All other parameters were adopted from the previous experiment. Figure 5 shows the reaction progress.

As the results show, decreasing the kinase concentrations substantially affects 2′3′-cGAMP synthesis. After 24 h, a concentration of 0.14 ± 0.01 mM 2′3′-cGAMP was reached. Obviously, ATP availability was limited for 2′3′-cGAMP synthesis. The ATP concentration remained low throughout the whole reaction progress with an average concentration of 0.03 ± 0.00 mM. ATP was obviously presumably consumed by *Sc*ADK, rather than being converted to the final product 2′3′-cGAMP by thscGAS. Thus, the thscGAS activity of 0.002 ± 0.001 U mg^−1^ was substantially lower compared to the previously achieved results (Figure S8). This demonstrates that precise adaptation is necessary for enzymatic reaction cascades, especially when two enzymes compete for one substrate. The K_M_ value of *Sc*ADK related to ATP is 100 ± 11 μmol L^−1^ and the K_M_ value of thscGAS related to ATP is 140 ± 4 μmol L^−1^ [28,34]. The *Sc*ADK has therefore just a slightly higher affinity towards ATP compared to thscGAS. The substantially higher activity of *Sc*ADK is probably responsible for decreased ATP availability for 2′3′-cGAMP synthesis. Interestingly, despite low 2′3′-cGAMP synthesis, the GTP concentration decreased from 2.03 ± 0.03 mM to 0.45 ± 0.04 mM within the first five hours. This proves that GTP is also consumed in other reactions of the cascade, such as in the initial phosphorylation of adenosine by *Sc*ADK.

To compare the results of the thscGAS cascade experiments, specific parameters were calculated and summarized in Table 2. The specific yield coefficients of 2′3′-cGAMP synthesis in relation to adenosine were determined. Enzyme usage takes the amount of product synthesized per amount of enzyme used into account.

As shown in Table 2, the reaction rate of 2′3′-cGAMP synthesis was the highest in the first experiment with a specific activity of 0.052 ± 0.014 U mg^−1^, although higher thscGAS concentrations were used in the subsequent experiments. This value is in the typical range for the intrinsic specific activity of thscGAS, demonstrating that the thscGAS-catalyzed reaction was running close to its optimum in these enzyme cascade conditions [25]. The specific thscGAS activity with changed enzyme ratios (50:5:50:120) was only 0.012 ± 0.004 U mg^−1^, which indicates that not enough ATP was provided by the cascade to fully exploit the activity of thscGAS. Additionally, the varying ratio of available ATP and GTP might have led to an inhibition, as it is known that the substrates compete with each other in the active site [34]. Equimolar supply of ATP and GTP results in the highest reaction rates for 2′3′-cGAMP synthesis. Nevertheless, the highest specific product yield coefficient was reached in this experiment, which was caused by an increased GTP supply with an initial concentration of 2.4 mM instead of 0.5 mM. The specific 2′3′-cGAMP yield related to GTP in this experiment was 0.40 ± 0.01 mol 2′3′-cGAMP per mol GTP. In screening studies with thscGAS, Rolf et al. achieved twice as high specific 2′3′-cGAMP yields related to GTP of 0.79 ± 0.24 mol 2′3′-cGAMP per mol GTP, which indicates the need for further optimization [25]. One possibility would be to re-phosphorylate the dephosphorylated GTP by suitable kinases to provide it again to the thscGAS-catalyzed reaction. In the last experiment with enzyme mass ratios of 5:0.5:5:120, the specific thscGAS activity was 0.002 ± 0.001 U mg^−1^. Here, only very low ATP concentrations were obtained, which obviously significantly limited 2′3′-cGAMP synthesis. Due to the large difference in activity between the *Sc*ADK and thscGAS, both of which compete for the substrate ATP, thscGAS was apparently limited to convert a sufficient amount to the product 2′3′-cGAMP. This demonstrates the need to provide excess ATP for an efficient 2′3′-cGAMP synthesis. Alternatively, the 2′3′-cGAMP synthesis rate could be increased by using another cGAS homologue as it is already known that the murine cGAS homolog has an almost fourfold higher activity of 0.279 U mg^−1^ and could thus lead to higher synthesis rates in this multi-enzyme cascade reaction [25].

The highest obtained product amount was 0.08 mol 2′3′-cGAMP per mol adenosine. Although this yield seems to be low, it might be a promising initial starting point for further optimization. It is worth mentioning that the chemical synthesis of 2′3′-cGAMP delivers yields of 5% within 3 days starting with phosphoramidite and it is therefore relatively costly and time-consuming [35,36]. Thus, 2′3′-cGAMP synthesis is in the same range based on the cheaper starting material adenosine.

In general, these results demonstrate the successful synthesis of 2′3′-cGAMP by an enzyme cascade starting from adenosine. This could presumably be further improved by replacing individual enzymes by more active enzymes. In addition, in a similar manner to the ATP synthesis cascade, GTP supply synthesized from guanosine by an enzyme cascade could be developed. Various PPK2s do not only synthesize ATP and its precursors but also GTP [37,38,39]. Furthermore, another promising optimization of the enzyme cascade could be the use of class III PPK2 enzymes, which enable us to synthesize nucleoside 5′-triphosphates from nucleoside 5′-monophosphates by a single enzyme [38]. Recently, the limited list of characterized PPK2 enzymes has been expanded with more than 50 new enzymes that can be used for ATP regeneration from AMP [40]. One of these enzymes, a class III PPK2 of an unclassified *Erysipelotrichaceae* bacterium, was used for ATP regeneration during the synthesis of 4-methoxybenzaldehyde on a multigram scale with a carboxylic acid reductase.

## 4. Conclusions

In this study, the synthesis of 2′3′-cGAMP was investigated by using a multi-enzyme cascade reaction with adenosine as initial substrate. It was shown that 2′3′-cGAMP can be synthesized in four enzyme-catalyzed reaction steps from adenosine as well as polyP and GTP in a biocatalytic one-pot reaction. The obtained yields of 0.08 mol 2′3′-cGAMP per mol adenosine are comparable to the classical chemical synthesis route starting from phosphoramidite. We have thus demonstrated the successful use of an enzyme cascade for the production of pharmaceutically relevant products. The cascade was iteratively optimized by adapting the enzyme concentrations and it turned out that slight changes led to a failing cascade, demonstrating the complexity of enzyme cascade optimization.

Since the cascade for ATP synthesis was successfully optimized, the obtained knowledge could be transferred to the synthesis of other nucleotides such as GTP with suitable polyphosphate kinases. The simultaneous synthesis of ATP and GTP from nucleosides would, however, increase the complexity of 2′3′-cGAMP synthesis. Nevertheless, sophisticated tuning of substrate, intermediate, and enzyme concentrations might enable to synthesize 2′3′-cGAMP from adenosine and guanosine. Consequently, further developments focus on establishing an enzymatic in situ substrate-supply for 2′3′-cGAMP synthesis, which could even circumvent the substrate inhibition of thscGAS by continuous nucleoside 5′-triphosphate supply at moderate concentrations.

In general, enzyme cascades have emerged as a widely used synthesis tool during recent years. However, the complexity of cascade reactions such as compatibility issues of the biocatalysts or cross-reactivities of intermediates delay their industrial application. Only a few enzyme cascade reactions are reported from industry with atorvastatin synthesis being the most prominent example [41,42]. Nevertheless, with careful development and optimization, the use of enzyme cascades could be further exploited for relevant applications and can thus contribute to an increasing importance in bioprocesses.

## Figures and Tables

**Table 1:** Reaction rates and specific activities of the enzymes *Sc*ADK, *Aj*PPK2, and *Sm*PPK2. The *Sc*ADK was tested with substrate concentrations of 10 mM adenosine and 10 mM ATP. The *Aj*PPK2 and *Sm*PPK2 were tested with substrate concentrations of 10 mM AMP and ADP, respectively, and 15 mM polyP calculated as single phosphate units. Assays were performed in 50 mM TRIS-HCl buffer containing 40 mM MgCl_2_ · 6H_2_O at pH 8.0 and 37 °C. Samples were taken after 0, 15, 30, 45, 60, 75, 90, 120, and 150 min. The assays were performed as biological duplicates.

Enzyme	Enzyme Concentration		Reaction Rate (μmol L^−1^ min^−1^)	Specific Activity (U mg^−1^)
	(mg L^−1^)	(nM)		
*Sc*ADK	5	130	21.7 ± 4.4	4.3 ± 0.9
*Aj*PPK2	0.5	9	34.6 ± 5.4	69.2 ± 10.8
*Sm*PPK2	5	140	13.8 ± 3.1	2.8 ± 0.6

**Table 2:** Reaction rates, specific activity, specific yield coefficients, and enzyme usage of the three thscGAS coupled cascade experiments. Reaction rates for 2′3′-cGAMP synthesis were determined from the first 5 h of reaction and used to calculate specific activities. The specific yield coefficients were determined from the initial and final concentrations of adenosine (Ado) and 2′3′-cGAMP. The enzyme usage describes the ratio between the obtained amount of 2′3′-cGAMP per mg enzyme.

*Sc*ADK:*Aj*PPK2: *Sm*PPK2:thscGAS Ratio		2′3′-cGAMP Reaction Rates (μmol L^−1^ min^−1^)	thscGAS Specific Activity (U mg^−1^)	Y2′3′-cGAMPAdo (mol_2′3′-cGAMP_ mol_Ado_^−1^)	Enzyme Usage (mg_2′3′__-cGAMP_ mg_enzyme_^−1^)
(mg:mg:mg:mg)	(µmol:µmol:µmol:µmol)				
50:50:50:40	1.3:0.9:1.4:0.7	2.10 ± 0.60	0.052 ± 0.014	0.030 ± 0.001	1.12 ± 0.04
50:5:50:120	1.3:0.09:1.4:2.1	1.42 ± 0.50	0.012 ± 0.004	0.070 ± 0.004	1.87 ± 0.19
5:0.5:5:120	0.13:0.009:0.14:2.1	0.25 ± 0.14	0.002 ± 0.001	0.077 ± 0.030	0.75 ± 0.03

## Data Availability

Data are contained within the article or Supplementary Materials.

## Supplementary Materials

The following are available online at https://www.mdpi.com/article/10.3390/biom11040590/s1, Figure S1: SDS gel of thscGAS purification; Figure S2: SDS gel of *Sc*ADK purification; Figure S3: SDS gel of *Aj*PPK2 purification; Figure S4: SDS gel of *Sm*PPK2 purification; Figure S5: SDS gel of protein purification of thscGAS, *Sm*PPK2, *Aj*PPK2, and *Sc*ADK; Figure S6–S8: Progress of 2′3′-cGAMP-synthesis during enzyme cascades reactions; Figure S9: Progress of AMP-synthesis by *Sc*ADK; Figure S10: Progress of ADP-synthesis by *Aj*PPK2; Figure S11: Progress of ATP-synthesis by *Sm*PPK2; Table S1: Retention times of HPLC measurements.
