# Supplementary material for: A Multi-Enzyme Cascade Reaction for the Production of 2′3′-cGAMP
Source: Biomolecules. 2021 Apr 16;11(4):590. doi: 10.3390/biom11040590 (PMC8073963; doi:10.3390/biom11040590)
Supplement: Supplementary file 1 [file biomolecules-11-00590-s001.zip › biomolecules-1187445-supplementary.pdf]

biomolecules

Supplementary Materials for: A Multi-Enzyme Cascade Reaction for the Production of 2‘3‘-cGAMP

Content

1. SDS gels of enzyme purification 1

2. Experimental data 4

1. SDS gels of enzyme purification

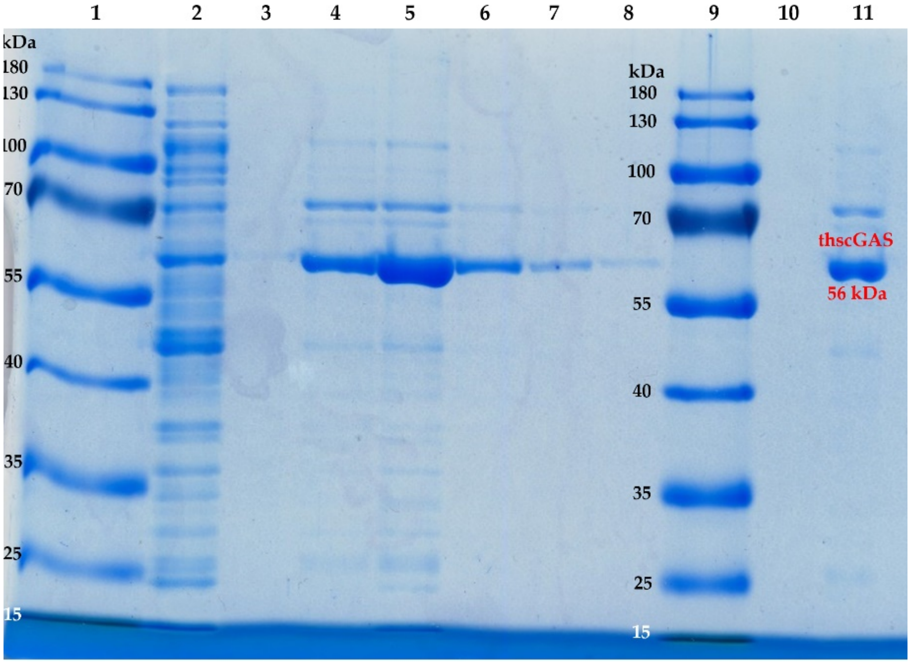

Figure S1. SDS gel of thscGAS purification. Lane **1, 9**: PageRuler™ #26616 (Thermo Fisher Scientific™, Waltham, MA, USA); **2**: Filtrate after cell disruption via sonication; **3:** Flow-through of IMAC column; **4–8**: Fractions 1 to 5 of protein elution of IMAC column; **10**: Flow-through of PD-10 column (GE Healthcare, Solingen, Germany); **11**: Purified thscGAS after buffer exchange, which is dissolved in 50 mM TRIS-HCl buffer (containing 40 mM $MgCl_2 \cdot 6H_2O$, pH 8).

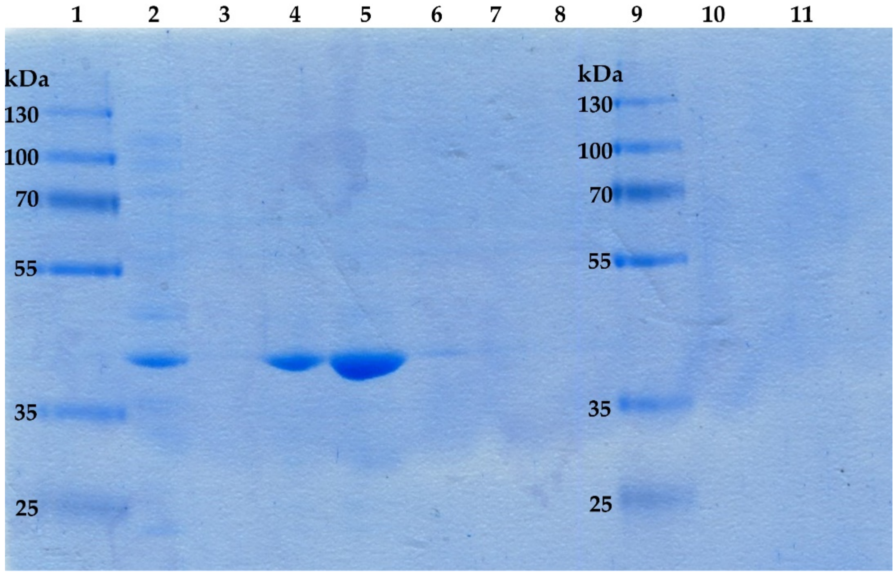

Figure S2. SDS gel of *Sc*ADK purification. Lane **1, 9**: PageRuler™ #26619 (Thermo Fisher Scientific™, Waltham, MA, USA); **2**: Filtrate after cell disruption via sonication; **3:** Flow-through of IMAC column; **4–8**: Fractions 1 to 5 of protein elution of IMAC column; **10**: Flow-through of PD-10 column (GE Healthcare, Solingen, Germany); **11**: Purified *Sc*ADK after buffer exchange, which is dissolved in 50 mM TRIS-HCl buffer (containing 40 mM $MgCl_2 \cdot 6H_2O$, pH 8).

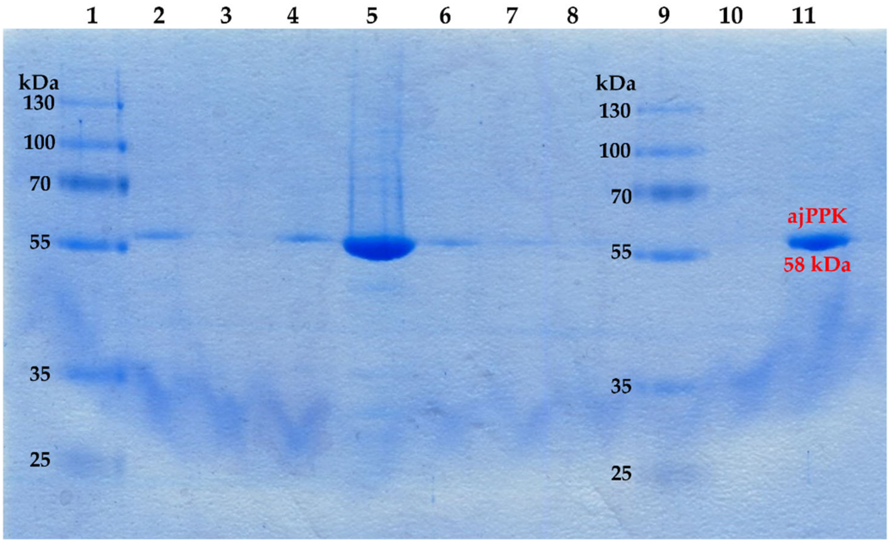

Figure S3. SDS gel of *Aj*PPK2 purification. Lane **1, 9**: PageRuler™ #26619 (Thermo Fisher Scientific™, Waltham, MA, USA); **2**: Filtrate after cell disruption via sonication; **3**: Flow-through of IMAC column; **4–8**: Fractions 1 to 5 of protein elution of IMAC column; **10**: Flow-through of PD-10 column (GE Healthcare, Solingen, Germany); **11**: Purified *Aj*PPK2 after buffer exchange, which is dissolved in 50 mM TRIS-HCl buffer (containing 40 mM $MgCl_2 \cdot 6H_2O$, pH 8).

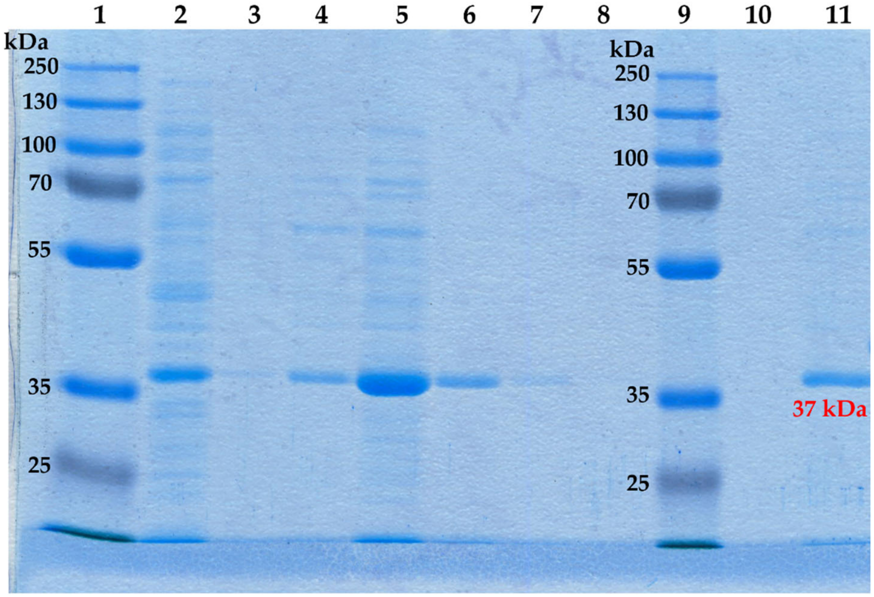

Figure S4. SDS gel of *Sm*PPK2 purification. Lane **1, 9**: PageRuler™ #26619 (Thermo Fisher Scientific™, Waltham, MA, USA); **2**: Filtrate after cell disruption via sonication; **3**: Flow-through of IMAC column; **4–8**: Fractions 1 to 5 of protein elution of IMAC column; **10**: Flow-through of PD-10 column (GE Healthcare, Solingen, Germany); **11**: Purified *Sm*PPK2 after buffer exchange, which is dissolved in 50 mM TRIS-HCl buffer (containing 40 mM $MgCl_2 \cdot 6H_2O$, pH 8).

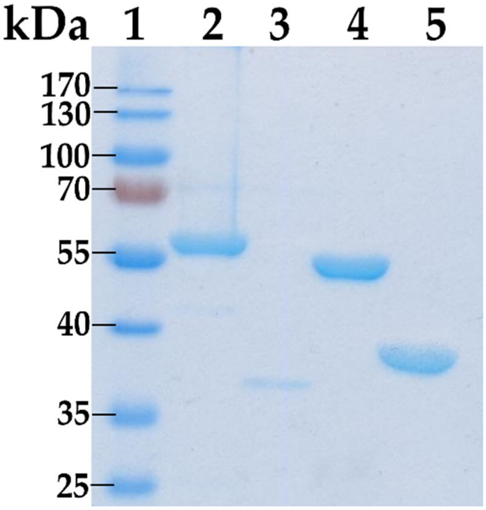

Figure S5. SDS gel of protein purification of thscGAS, *Sm*PPK2, *Aj*PPK2, and *Sc*ADK. Lane **1**: PageRuler™ #26616 (Thermo Fisher Scientific™, Waltham, MA, USA); **2**: Purified thscGAS; **3**: purified *Sm*PPK2; **4**: Purified *Aj*PPK2; **5**: purified *Sc*ADK. All enzyme samples were taken after buffer exchange. The enzymes are dissolved in 50 mM TRIS-HCl buffer (containing 40 mM $MgCl_2 \cdot 6H_2O$, pH 8). The enzymes purified here were used for the enzyme cascades with the *Sc*ADK:*Aj*-PPK:*Sm*PPK:thscGAS mass ratio 50:5:50:120 and 5:0.5:5:120.

2. Experimental data

Table S1. Retention times of the analytes in HPLC measurements.

Retention times [min]	Analytes
3.47	GTP
6.45	ATP
7.32	ADP
8.35	AMP
8.83	2'3'-cGAMP
11.58	Adenosine

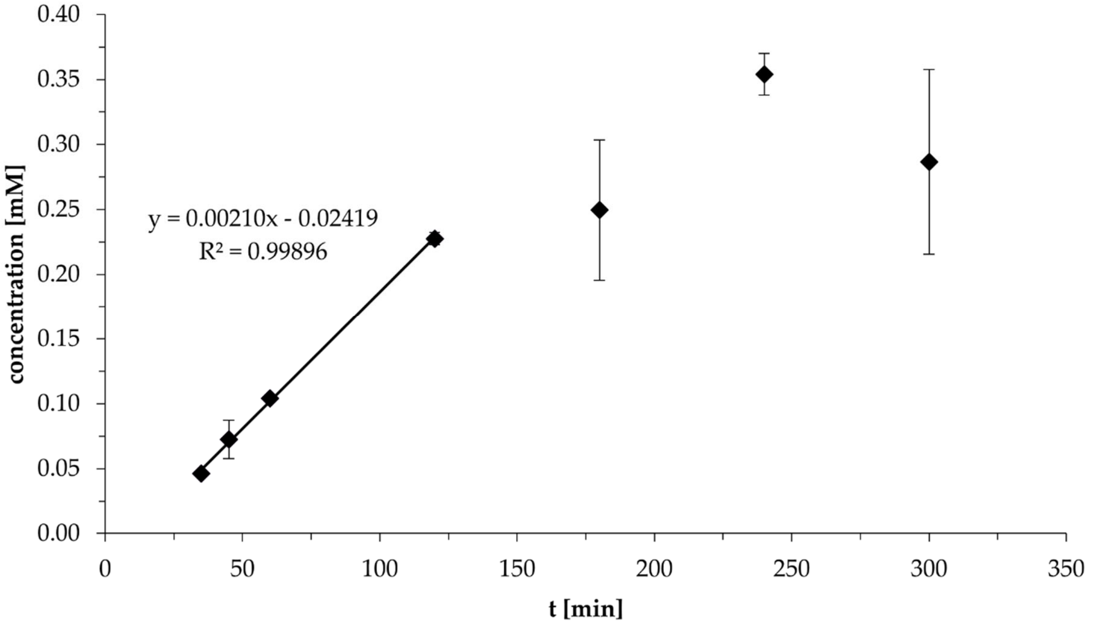

Figure S6. Progress of cGAMP-synthesis within the first 5 h with the used enzyme concentrations: 50 mg L^{-1} *Sc*ADK, 50 mg L^{-1} *Aj*PPK2, 50 mg L^{-1} *Sm*PPK2, and 40 mg L^{-1} thscGAS.

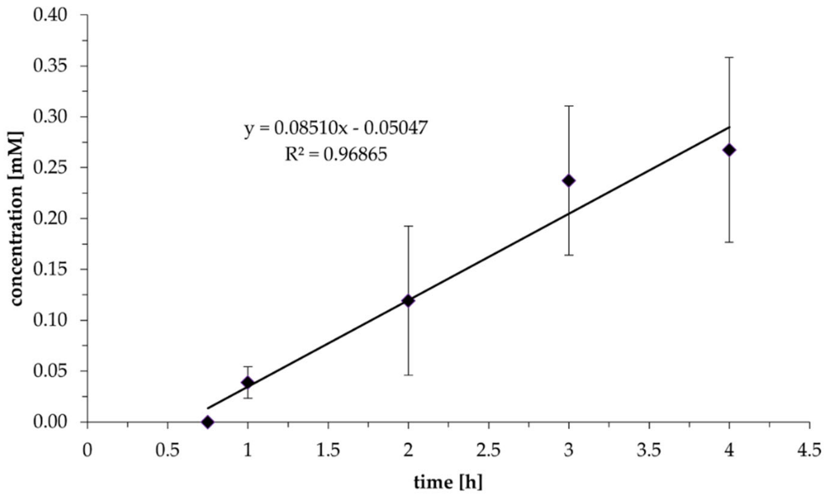

Figure S7. Progress of cGAMP-synthesis within the first 4 h with the used enzyme concentrations: 50 mg L^{-1} *Sc*ADK, 5 mg L^{-1} *Aj*PPK2, 50 mg L^{-1} *Sm*PPK2, and 120 mg L^{-1} thscGAS.

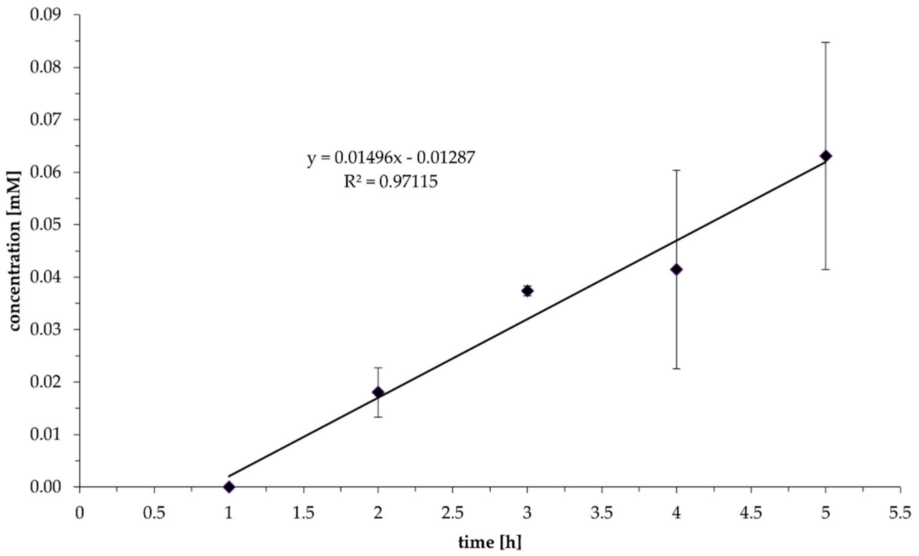

Figure S8. Progress of cGAMP-synthesis within the first 5 h with the used enzyme concentrations: 5 mg L^{-1} *Sc*ADK, 0.5 mg L^{-1} *Aj*PPK2, 5 mg L^{-1} *Sm*PPK2, and 120 mg L^{-1} thscGAS.

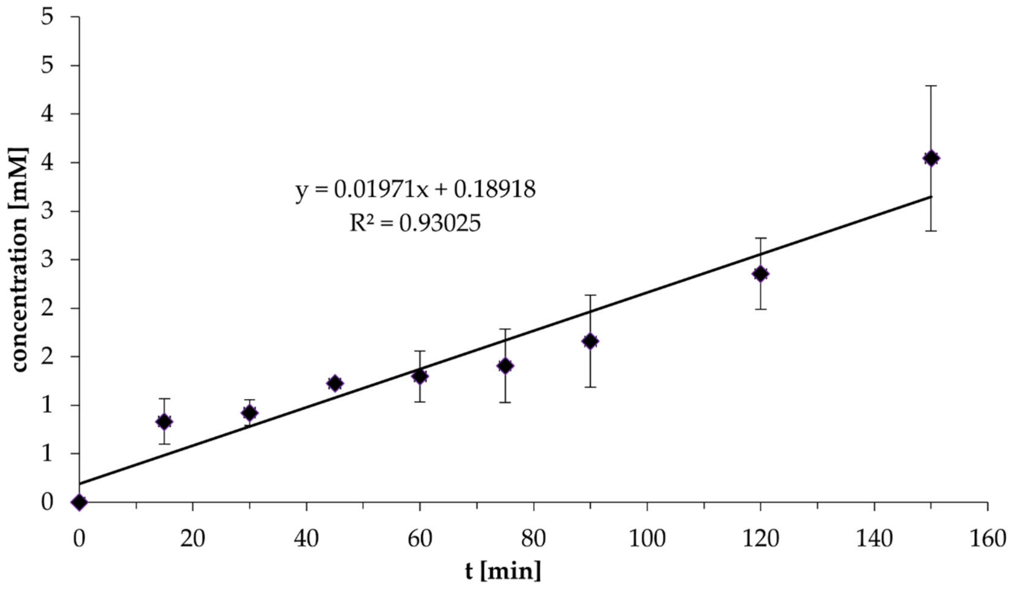

Figure S9. Progress of AMP-synthesis by *Sc*ADK within the first 2.5 h. Used concentrations: 5 mg L^{-1} *Sc*ADK, 10 mM adenosine, and 10 mM ATP.

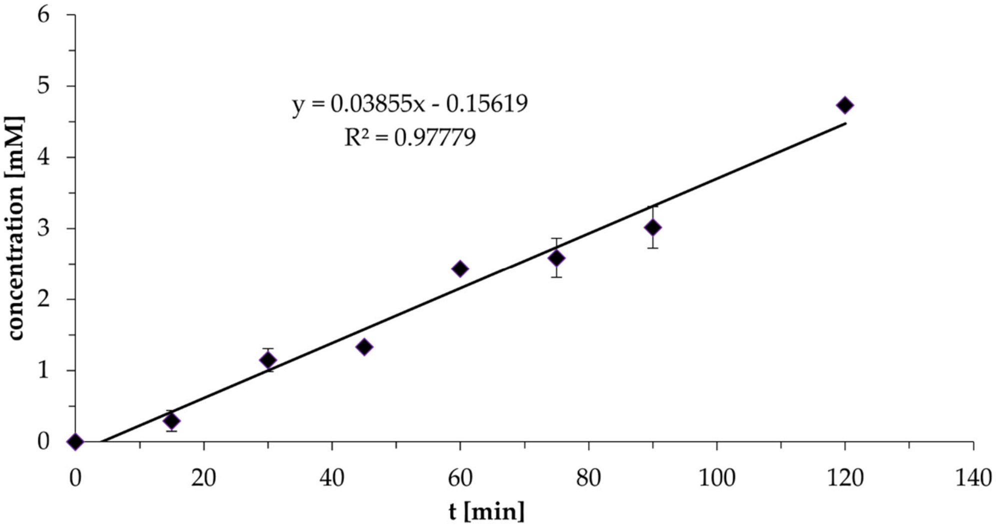

Figure S10. Progress of ADP-synthesis by *Aj*PPK2 within the first 2 h. Used concentrations: 0.5 mg L^{-1} *Aj*PPK2, 10 mM AMP, and 15 mM polyP.

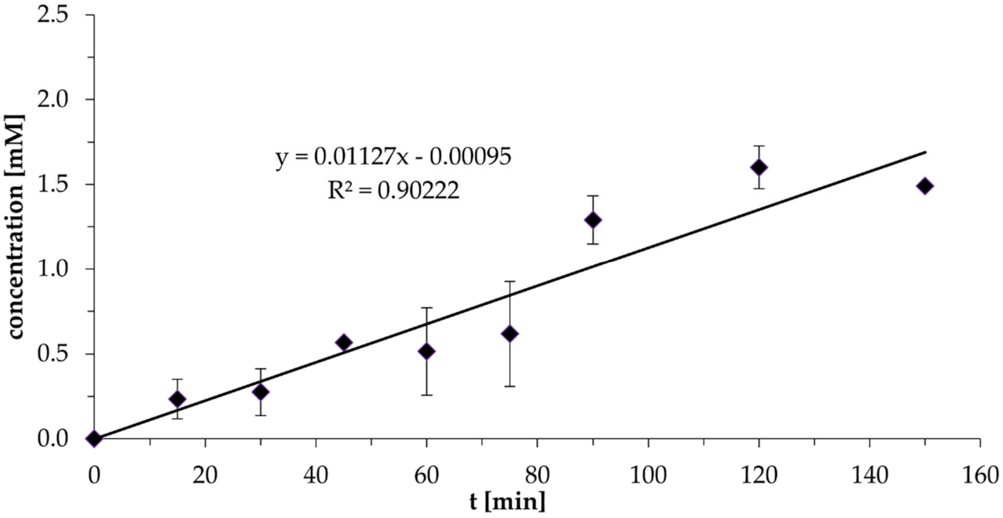

Figure S11. Progress of ATP-synthesis by *Sm*PPK2 within the first 2.5 h. Used concentrations: 5 mg L^{-1} *Sm*PPK2, 10 mM ADP, and 15 mM polyP.
